# Hair Follicle Transcriptome Analysis Reveals Differentially Expressed Genes That Regulate Wool Fiber Diameter in Angora Rabbits

**DOI:** 10.3390/biology12030445

**Published:** 2023-03-14

**Authors:** Dongwei Huang, Haisheng Ding, Yuanlang Wang, Guanglong Cheng, Xiaofei Wang, Taishan Leng, Huiling Zhao

**Affiliations:** Anhui Provincial Key Laboratory of Livestock and Poultry Product Safety Engineering, Institute of Animal Husbandry and Veterinary Medicine, Anhui Academy of Agricultural Sciences, Hefei 230031, China; hdwscience@163.com (D.H.); dinghs123@163.com (H.D.);

**Keywords:** wool fiber diameter, hair follicles, RNA-Seq, GSEA, PPI, hair structure

## Abstract

**Simple Summary:**

Wool fiber diameter (WFD) is one of the most important economic traits of wool, especially in Angora rabbits. Emerging studies have been conducted on sheep and goats. However, few studies of WFD have been reported in rabbits. In our study, the diameter of coarse and fine wool and their respective hair follicles in Wan strain Angora rabbits were analyzed, and it was found that the diameter of coarse wool and hair follicles was larger than that of fine wool and hair follicles. High-throughput sequencing analysis was performed using hair follicles from coarse and fine wool. Differentially expressed genes (DEGs), including transcription factors, keratin-associated protein (KAP) and keratin (KRT) families, and extracellular matrix (ECM)-related genes, were screened. Enrichment analysis showed that skin development, epidermal cell and keratinocyte differentiation, epithelium development, and Notch and ribosome signaling pathways were important in regulating hair structure. Gene set enrichment analysis (GSEA) and protein–protein interaction (PPI) analysis further identified six important signaling pathways and functional DEGs, including *LEF1*, *FZD3*, *SMAD3*, *ITGB6*, and *BMP4*. The results provide valuable data for screening molecular markers involved in hair structure and facilitating enhanced selection of super-fine wool rabbits through gene-assisted selection.

**Abstract:**

Wool fiber diameter (WFD) is an important index of wool traits and the main determinant of wool quality and value. However, the genetic determinants of fiber diameter have not yet been fully elucidated. Here, coarse and fine wool of Wan strain Angora rabbits and their hair follicle traits were characterized. The results indicated significant differences in the diameters of wool fibers and their hair follicles. The RNA sequencing (RNA-Seq) technique was used to identify differences in gene expression in hair follicles between coarse and fine wool. In total, 2574 differentially expressed genes (DEGs) were found between the two hair follicle groups. Transcription factors, keratin-associated protein (KAP) and keratin (KRT) families, and ECM-related genes may control the structure of fine fibers in rabbits. Gene ontology (GO) and Kyoto Encyclopedia of Genes and Genomes (KEGG) analyses revealed that skin development, epidermal cell and keratinocyte differentiation, epithelium development, and Notch and ribosome signaling pathways were significantly enriched, respectively. GSEA further filtered six important pathways and related core genes. PPI analysis also mined functional DEGs associated with hair structure, including *LEF1*, *FZD3*, *SMAD3*, *ITGB6*, and *BMP4*. Our findings provide valuable information for researching the molecular mechanisms regulating wool fiber and could facilitate enhanced selection of super-fine wool rabbits through gene-assisted selection in the future.

## 1. Introduction

Rabbits are small domestic animals that provide not only meat and fur but also wool, which is a raw material [1] that is extremely important for the textile industry [2]. Wool quality traits such as length, coarse wool rate, and fiber diameter are important goals in rabbit breeding programs. Wool fiber diameter (WFD) determines 75% of the value of wool fibers [3]. The WFD variation-induced profit accounts for 61% of the total profit of wool [4]. WFD is an important economic indicator of Angora rabbits and it determines processing performance, end-use of wool, and wool quality and value [2,5]. Since the 1980s, the coarse wool of Angora rabbits was the favorite of the international Angora rabbit market. During the past two decades, the demand for the fine wool of rabbits has been gradually increasing owing to improvements in wool spinning technology and the development of rabbit wool fabric.

Wool fibers are formed by keratinocytes which derive from progenitor populations at the base of the hair follicle [6]. Rabbit wool is divided into coarse and fine fibers according to the fiber diameter. Rabbit skin has two different types of hair follicles: the primary hair follicle (PHF) producing coarse hair, and the secondary hair follicle (SHF) producing fine hair [7,8,9]. The follicle diameter of PHFs is much larger than that of SHFs in Cashmere goats [10]. Most studies of WFD have focused on sheep and goats [3,11,12,13,14]. However, studies on the mechanisms underlying WFD in rabbits are limited.

Hair follicles form the basis for hair growth. At least 20 different cells and tissues, including the dermal papilla, inner root sheath, outer root sheath, and hair matrix, are involved in the process of cell differentiation and hair follicle development [15]. Dermal papillary cells at the base of the hair follicle are a population of mesenchymal cells, that provide signals to specify the size and shape of the wool [16,17]. It has been reported that WFD is strongly related to the size of the dermal papilla cells and matrix in mammals [3,6,18,19,20], and is largely determined in the course of hair follicle growth and morphogenesis [21]. In addition, many genes, including distal-less homeobox 3 (*Dlx3*), lymphatic enhancer factor 1 (*Lef1*), epidermal growth factor (*EGF*), fibroblast growth factor 5 (*FGF5*), insulin-like growth factor-1 (*IGF-I*), and Wnt, are involved in regulating hair follicle development [22,23,24,25,26].

Gene expression profile analysis plays a vital role in the discovery and prediction of gene function. Gene expression profile analysis showed functional genes related to fur color determination using Solexa sequencing in rabbits [27]. Differentially expressed genes regulating fur development were obtained by RNA-Seq analysis in rex rabbits [28]. However, limited data are available on the expression information of specific genes in hair follicle tissues that reflect the wool fiber diameter in Angora rabbits. The present study used the RNA-Seq-based approach to identify the DEG-regulated wool fiber diameter in Angora rabbits in an attempt to identify novel functional genes related to wool fineness.

## 2. Materials and Methods

### 2.1. Animals

The rabbits used in the experiments were obtained from the Animal Husbandry Institute of the Anhui Academy of Agriculture Sciences and raised in the same conditions, fed to appetite, and drinking water was supplied by an automatic water feeder.

### 2.2. Fiber Samples and Diameter Measurement

Fiber samples were obtained from three healthy, one-year-old Wan strain Angora rabbits. The wool fibers were collected from the back of the body by the roots and pulled out. Then, the hair follicles of fibers were cut off and placed in a 2.0 mL freezing tube and immediately frozen in liquid nitrogen. Fiber samples were used for diameter measurements. The wool fiber diameter was measured using W1Vnt software according to the manufacturer’s instructions.

### 2.3. Skin Sample Collection and Histological Examination

For sample collection, anesthesia through an ear vein injection of 0.7% pentobarbital sodium (6 mL/kg) was first administered. Then, skin tissue samples were collected from the backs of rabbits. Histological analysis was performed as previously described [29,30].

### 2.4. cDNA Library Construction and Sequencing

Total RNA was extracted from hair follicle samples using TRIzol reagent (Invitrogen, Carlsbad, CA, USA), following the manufacturer’s instructions. Then, the NanoPhotometer spectrophotometer (Implen, Westlake Village, CA, USA) and Agilent 2100 bioanalyzer (Agilent Technologies, Santa Clara, CA, USA) were used to determine the RNA purity, concentration, and integrity. The high-quality RNA for cDNA library construction was obtained after RNA sample quality control. Then, six libraries for hair follicles of coarse and fine wool were constructed using the NEBNext^®^ Ultra™ RNA Library Prep Kit for Illumina^®^ (NEB, Ipswich, MA, USA), quantified with a Qubit2.0 fluorometer. Library insert size was determined using an Agilent 2100 bioanalyzer, and sequenced using the Illumina NovaSeq 6000 platform (Illumina, USA).

### 2.5. Transcriptome Mapping and Differential Gene Expression Analysis

The low-quality reads, adaptor sequences, and N reads were removed from raw sequencing reads using *fastp* (version 0.19.7). The cleaned, high-quality reads from hair follicles of coarse and fine wool were aligned against the *Oryctolagus cuniculus* genome using HISAT2 [31]. Unique-mapped reads were used for further analysis. Transcripts were assembled using stringtie (version 1.3.3b), and gene expression was quantified using featureCounts (version 1.5.0-p3) [32]. The value of the kilobase of transcript per million fragments mapped (FPKM) was used to estimate the gene expression levels in hair follicle samples [33]. DESeq2 was used to perform differential expression analysis [34]. Differentially expressed genes (DEGs) were filtered using the standards log_2_|fold change| ≥1 and padj ≤ 0.05. GO terms (http://geneontology.org, accessed on 2 August 2022) [35] and KEGG pathway (http://www.genome.jp/kegg/, accessed on 2 August 2022) [36] were also annotated.

### 2.6. GSEA

All genes in hair follicles of coarse and fine wool were applied using GSEA with GSEA software (http://www.broadinstitute.org/gsea/index.jsp, accessed on 8 November 2022). Enrichment scores of gene sets were calculated using full ranking, then the distribution of gene sets was obtained in the list. A NOM *p*-value < 0.05 and |NES| > 1 were considered to indicate significant enrichment.

### 2.7. Establishment of PPI Network

The PPI network was constructed using the STRING (version 11.0) [37] online database. PPIs with a confidence score < 0.7 and disconnected nodes were discarded. The network was visualized and analyzed using Cytoscape software (version 3.8.2) (http://www.cytoscape.org/, accessed on 16 December 2022).

### 2.8. Validation of RNA-Seq

Nine candidate genes related to hair structure were selected from DEGs for validation by quantitative real-time PCR (qRT-PCR). The qRT-PCR was performed on an ABI 7500 (Applied Biosystems, Waltham, MA, USA) using TransStart Green qRT-PCR SuperMix (Transgen, Beijing, China). The primer sequences for the qRT-PCR are in Supplementary File Table S1. The amplification conditions were referred to in our previous studies [29]. Quantitative variation and relative fold changes were estimated using the 2^−ΔΔCT^ method [38], and the rabbit glyceraldehyde-3-phosphate dehydrogenase (*GAPDH*) gene was used as an internal control. The qRT-PCR analysis was conducted at least in triplicate.

### 2.9. Statistical Analyses

The data were presented in terms of the mean ± standard deviation (SD) and were determined using GraphPad Prism 5. Significant differences were estimated using Student’s *t*-test. *p* values < 0.05 were considered significant.

## 3. Results

### 3.1. Characterization of Coarse and Fine Wool and Hair Follicle Traits of Angora Rabbits

**Figure 1:**
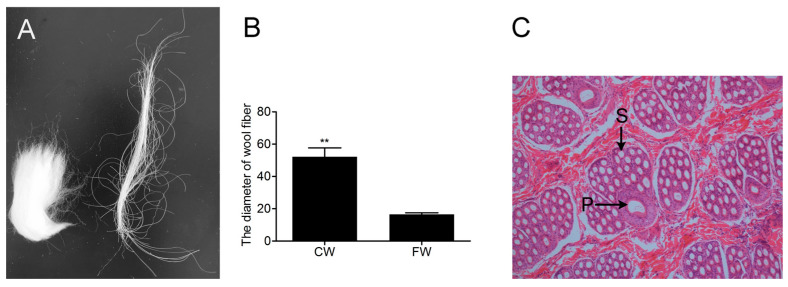
Comparison of coarse and fine wool and hair follicle traits of Wan strain Angora rabbits. (**A**) The appearance of coarse and fine wool. (**B**) The diameter of coarse and fine wool. ** *p* < 0.01. (**C**) Histological analysis of hair follicles. P means primary hair follicle; S means secondary hair follicle.

To compare the morphological traits between coarse and fine wool of Angora rabbits, we collected wool samples and recorded fiber diameter (Figure 1A,B). The results revealed that there were significant differences in diameter between the two types of wool (*p* < 0.01) (Figure 1B). The morphology of the PHF and SHF, which produce coarse and fine wool, respectively, is shown in Figure 1C. The results indicate marked differences in fiber diameter and hair follicle morphology between coarse and fine wool in Angora rabbits.

### 3.2. Illumina Transcriptome Sequencing of Hair Follicles

**Figure 2:**
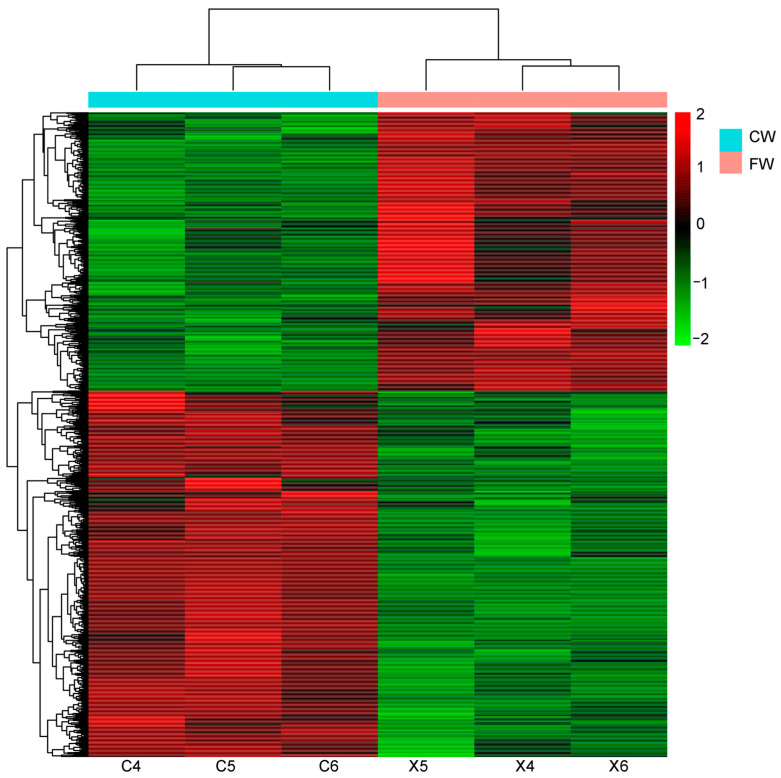
Heatmap analysis of the DEGs in hair follicles between coarse and fine wool from rabbits. Increased expression is marked in red, and decreased expression is marked in green; C represents coarse wool group; X represents fine wool group.

To comprehensively understand the transcriptome profiles that could be involved in regulating fiber diameter differences, hair follicle transcriptomes of coarse and fine wool of Angora rabbits were examined. We obtained 45.8–47.4 million raw reads and 43.3–44.4 million high-quality clean reads, and Q30 was ≥93.50% (Supplementary File Table S2). The total number of clean reads mapped to the rabbit genome was greater than 87%, and the map rates of unique reads were ≥74.96% (Supplementary File Table S2). After normalization, a total of 2574 DEGs (|log_2_FoldChange| ≥ 1 and false discovery rate padj ≤ 0.05) were analyzed between the groups, comprising 1462 genes that were more highly expressed in hair follicles of coarse wool and 1112 genes that were more highly expressed in hair follicles of fine wool (Supplementary File Figure S1). Hierarchical clustering showed that the gene expression patterns in the coarse wool group were different from those in the fine wool group (Figure 2). Transcription factors, including *DlX3*, *LEF1*, msh homeobox 2 (*MSX2*), and forkhead box N1 (*FOXN1*) were found to be highly expressed in the hair follicles of fine wool from Angora rabbits. A number of genes were identified as DEGs from the KRT and KAP families, including *KRT71*, *KRT72*, *KRT73*, *KRT17*, *KRT25*, *KRT10*, *KRT82*, *KRT35*, *KRT85*, *KRT5*, *KRT32*, *KRT34*, *KRT39*, *KRT77*, *KRTAP6-1*, *KRTAP3-1*, and *KRTAP17-1* (Table 1). Of these, 11 genes were highly expressed in the fine wool group. In addition, ECM-related genes (*COL17A1*, *COL11A1*, *COL15A1*, and *COL1A2*) were differentially expressed between the two groups. The results suggested that transcription factors, KRT and KAP families, and ECM-related genes were key candidates in determining the structure of fine fibers in rabbits.

### 3.3. Functional Annotation of DEGs from Hair Follicles of Coarse and Fine Wool

**Figure 3:**
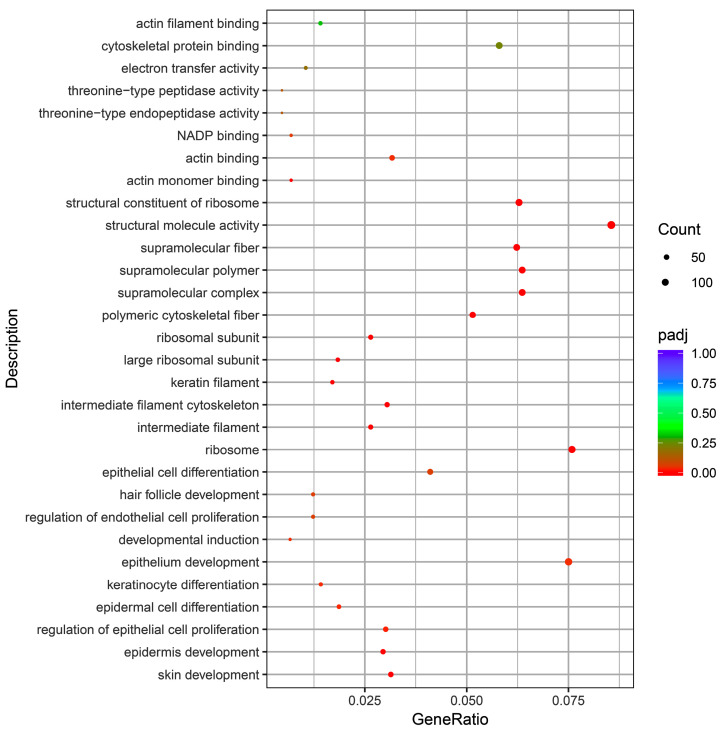
GO analysis of DEGs in hair follicles between coarse and fine wool. *p* value < 0.05 was considered to indicate a significant difference.

**Figure 4:**
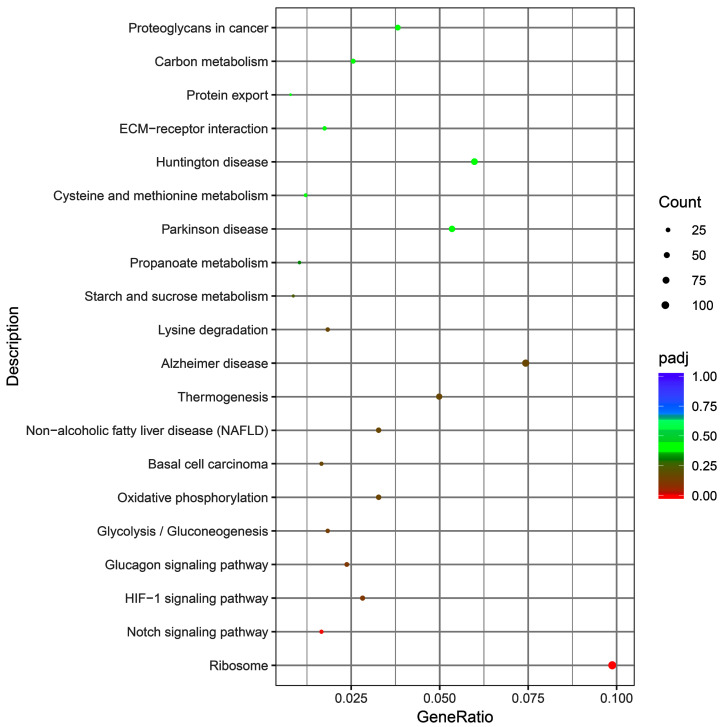
The enriched KEGG pathways of DEGs in hair follicles between coarse and fine wool. *p* value < 0.05 was considered to indicate a significant difference.

GO and KEGG were used to identify the potential functions of the DEGs. The GO terms and KEGG pathways with padj ≤ 0.05 were considered to be significantly enriched. In addition, 1545 and 1095 DEGs were involved in 3748 biological processes based on GO terms and 316 KEGG pathways, respectively. For GO term analysis, a comparison between hair follicles of coarse and fine wool showed that some biological processes, including skin development (50 DEGs), epidermis development (47 DEGs), regulation of epithelial cell proliferation (48 DEGs), epidermal cell differentiation (30 DEGs), keratinocyte differentiation (23 DEGs), epithelium development (118 DEGs), and developmental induction (11 DEGs) (Figure 3, Supplementary File Table S3), were substantially enriched. In addition, the Notch and ribosome signaling pathways were substantially enriched by DEGs between the hair follicles of coarse and fine wool (Figure 4). This suggests that DEGs in these GO categories and KEGG pathways may play key roles in regulating wool traits.

### 3.4. GSEA Identifies Hair Structure-Associated Signaling Pathways

**Figure 5:**
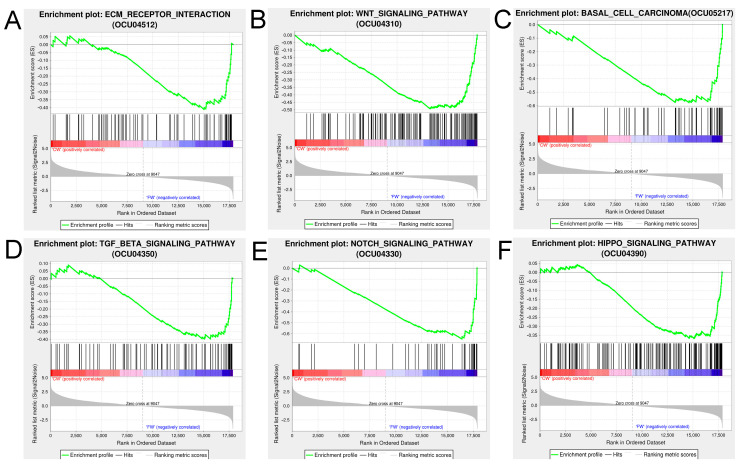
Gene set enrichment analysis was used to analyze the substantially enriched signaling. The enrichment scores of genes in the pathways were calculated using the GSEA algorithm. NOM *p*-value < 0.05 and |NES| > 1 were considered significant enrichment. (**A**) Extracellular matrix receptor interaction. (**B**) Wnt signaling. (**C**) Basal cell carcinoma. (**D**) TGF-β signaling. (**E**) Notch signaling. (**F**) Hippo signaling.

We further investigated the differences in gene expression levels in hair follicles between the coarse and fine wool groups and explored the function of the wool fiber diameter-related signal transduction pathway through GSEA. Significantly enriched signaling pathways were obtained based on their normalized enrichment scores and core related genes were identified. The results showed that six signaling pathways were substantially enriched in the fine wool group: ECM receptor interaction (*LAMA1*, *COL4A6*, *COL1A1*, *ITGB6*, *HSPG2*, *COL4A1*, and *COL4A2*), Wnt signaling (*RSPO3*, *WNT1*, *GSK3B*, *WNT9B*, *FZD7*, *CUL1*, *SFRP2*, *PPARD*, and *WNT16*), basal cell carcinoma (*WNT16*, *GLI2*, *WNT1*, and *FZD4*), TGF-β signaling (*AMHR2*, *INHBA*, *LTBP1*, and *ZFYVE16*), Notch signaling (*HES1*, *NCSTN*, *DVL2*, *DTX2*, and *RBPJ*), and Hippo signaling (*STK3* and *TGFBR1*) (Figure 5, Table 2, Supplementary File Tables S4–S9). Of the core genes, only *ITGB6* was differentially expressed in the coarse and fine wool groups.

### 3.5. Analysis Results of PPI

**Figure 6:**
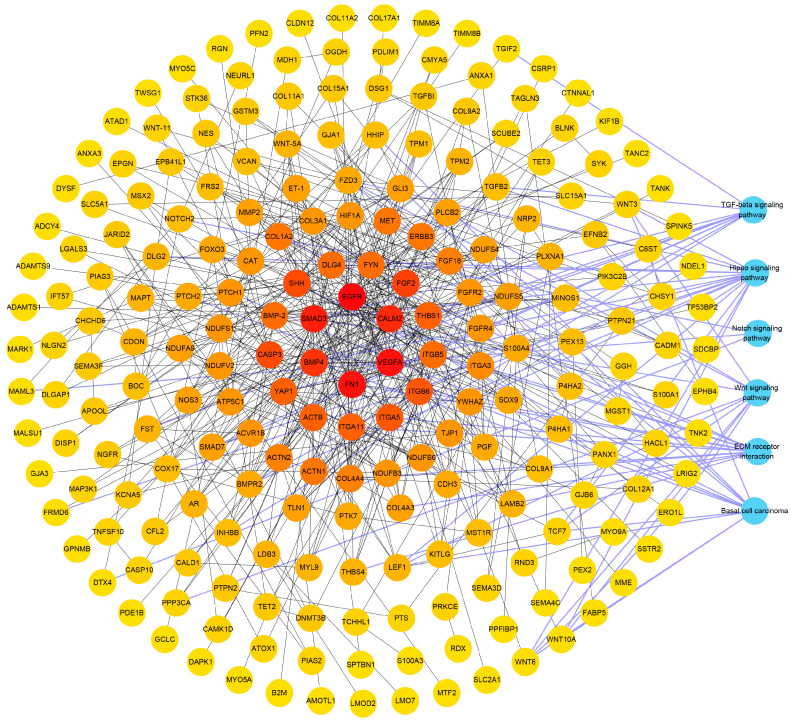
PPI analysis for DEGs in hair follicles between the coarse and fine wool groups. Each node means a gene, and each edge means the interaction between genes. The blue nodes mean the signaling pathways enriched by DEGs. A darker color indicates a stronger interaction with more genes.

To further explore the mechanism of DEGs involved in regulating hair structure and establish the interaction between differential genes, a PPI network based on DEGs was constructed using the STRING database and Cytoscape software for functional association analysis (Figure 6). A total of 219 genes formed a tightly connected network with 490 edges in the coarse and fine hair follicles, of which, the hub node epidermal growth factor receptor (*EGFR*) had the most connections owing to its interactions with 30 genes in the network, followed by the vascular endothelial growth factor A (*VEGFA*) and fibronectin 1 (*FN1*), which had 20 connections. In addition, the DEGs with high degree values (including *FN1*, *ITGB6*, *COL1A2*, *ITGA11*, *LAMB2*, *ITGA5*, *ITGA3*, and *ITGB5)* were involved in ECM–receptor interaction; *LEF1*, *FZD3*, *PLCB2*, and *SMAD3* were involved in Wnt signaling; *LEF1*, *FZD3*, *PTCH2*, *GLI3*, *PTCH1*, *SHH*, and *BMP4* were involved in basal cell carcinoma; *FST*, *BMPR2*, *SMAD7*, *ACVR1B*, *THBS1*, *BMP4*, and *SMAD3* were involved in TGF-β signaling; *NOTCH2*, *MAML3*, and *DTX4* were involved in Notch signaling; and *LEF1*, *DLG2*, *FZD3*, *BMPR2*, *SMAD7*, *YWHAZ*, *DLG4*, *YAP1*, *BMP4*, and *SMAD3* were involved in the Hippo signaling pathway. This suggests that the high-degree nodes are potential candidate genes regulating hair diameter.

### 3.6. Validation of DEGs with qRT-PCR

**Figure 7:**
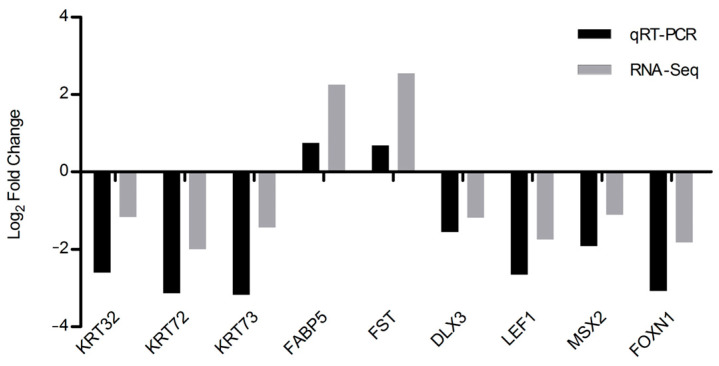
Validation of the RNA-Seq results of nine DEGs with qRT-PCR.

To evaluate our RNA-Seq results, *KRT32*, *KRT72*, *KRT73*, *FABP5*, *FST*, *DLX3*, *LEF1*, *MSX2*, and *FOXN1* were selected and their expression patterns in the hair follicles of coarse and fine wool of Angora rabbits were examined using qRT-PCR (Figure 7). *FABP5* and *FST* were upregulated in the hair follicles of the coarse wool groups; *KRT32*, *KRT72*, *KRT73*, *DLX3*, *LEF1*, *MSX2*, and *FOXN1* were upregulated in the hair follicles of the fine wool groups. The results showed that the trends in expression of these genes were similar to those of the RNA-Seq, demonstrating our analysis was reliable.

## 4. Discussion

Wool fiber diameter is commercially important in fiber-producing animals because of its effects on performance during texturing [13]. Rabbit hair is one of the most favored natural fibers used in textile industries, therefore breeding fine wool rabbits is a target of Angora rabbit breeding industries. Secondary hair follicles of fine wool Angora rabbits are the main hair follicles and are key determinants of the mean fiber diameter and rabbit wool characteristics. The heritability of fiber diameter is 0.59, which is relatively high [39]. However, the major genes underlying wool traits in Angora rabbits remain unclear. Therefore, we sought to generate a comprehensive list of the genes involved in regulating the fiber diameter of Angora rabbits.

There are two types of hair follicles: PHFs produce coarse hair in mammals, and SHFs produce fine hair [8,40]. The most obvious morphological difference between PHFs and SHFs is the follicle size. We compared the morphological structure differences between coarse and fine wool and the histological differences in the hair follicles of coarse and fine wool from Wan strain Angora rabbits. The diameter of coarse wool was substantially larger than that of fine wool, which is consistent with the histological results of the hair follicles of the two types of fibers. The histological results showed that the follicle diameter of the PHF was much larger than that of the SHF in rabbits, which was consistent with the result in goats [10].

We also investigated the genetic mechanism concerning the development of coarse and fine fibers by sequencing the transcriptomes of hair follicles and mapping the reads to rabbit genome assembly and annotated genes. The mRNA expression profiles of the hair follicles of coarse and fine wool were compared, and 2574 DEGs were obtained. Important pathways and transcription factors play critical roles in initiating and promoting differentiation of the matrix cells, including bone morphogenetic protein (BMP) and Notch signaling and transcription factors such as *MSX2* and *FOXN1*, which are required for hair shaft differentiation [41,42]. *FOXN1* is required for proper assembly of the hair medulla, and dysfunction of the *FOXN1* gene in mice causes severe developmental defects of the thymus and a hairless phenotype [43,44]. *DLX3* plays a crucial role in regulating hair follicle differentiation and regeneration, and it controls inner root sheath and hair shaft differentiation [45]. *DLX3* and *LEF1* may promote the maturation of SHFs [22]. Four important transcription factors associated with hair follicle development were highly expressed in the hair follicles of the fine wool group, suggesting that they have important roles in determining the structure of fine fibers in Angora rabbits.

KRTs and KAPs determining the quality of the fiber are the main structural proteins of wool fibers [8]. KAPs may be important for altering hair structure and diameter [46]. In total, we detected 14 differentially expressed KRT genes and 3 differentially expressed KAP genes, of which 16 genes were detected with FPKM>1000 in both types of follicles. The KAP proteins have previously been assigned to three groups: high-sulfur, ultrahigh-sulfur, and high-glycine–tyrosine [47]. *KRTAP3-1* and *KRTAP17-1* were in the high-sulfur and ultrahigh-sulfur group, respectively, and highly expressed in the hair follicles of fine wool. The results suggest that the proteins are important for the formation of fine wool. In addition, nine genes were selected for validation using qRT-PCR, and the results matched the RNA-Seq data, demonstrating the reliability of our sequencing data.

GO functional enrichment analysis showed that a large number of DEGs were involved in regulating skin development, epidermis development, epidermal cell differentiation, regulation of epithelial cell proliferation, keratinocyte differentiation, and epithelium development. The results indicated that DEGs are potential regulators of hair structure and diameter in rabbits. KEGG enrichment analysis is often used for gene function study. Interactions between the Wnt, Notch, Hedgehog, and BMP signaling pathways are involved in regulating normal hair follicle development [48]. In the present study, the Notch and ribosome pathways were substantially enriched with DEGs. Notch signaling is known to regulate cell fate, proliferation, differentiation, and pattern formation during the postnatal development of hair follicles [49,50]. Notch signaling is also necessary for complete maturation of hair follicles and postnatal hair cycle homeostasis [1,51,52]. It has been reported that DEGs between black and white hair follicles in giant pandas are also enriched in the ribosomal pathway [53]. Our results demonstrated that the Notch and ribosome pathways were key pathways of fiber structure. The GO functional enrichment and pathway analysis of DEGs in the current study elucidate the mechanisms of hair follicle development and the difference in fiber structure between coarse and fine wool in rabbits.

We used the GSEA approach [54] to compare biological differences in all gene expression patterns in hair follicles between the coarse and fine wool groups. Based on the KEGG-based list, all genes with higher expression in the fine wool group were substantially enriched in pathways related to ECM–receptor interaction, Wnt signaling, TGF-β signaling, basal cell carcinoma, Notch signaling, and Hippo signaling. Canonical pathways such as the Wnt, Hippo, and TGF-β signaling pathways have been proven to be closely associated with hair growth [55]. The ECM–receptor interactions play a vital role in regulating the hair follicle size. ECM is widely present in the dermal sheath and papilla [56]. The amount of ECM per cell determines the volume of the dermal papilla, and the volume of the dermal papilla determines the size of the hair follicle [57]. WFD is significantly associated with the size of dermal papilla cells [19,20]. The core genes in the pathway may be involved in regulating WFD, including *LAMA1*, *COL4A6*, *COL1A1*, *ITGB6*, *HSPG2*, *COL4A1*, and *COL4A2*. The *ITGB6* gene was expressed differently between the two groups, and it is a candidate gene for hairlessness and skin with few hair follicles [58]. The basal cell carcinoma pathway is also considered to be associated with hair growth [29]. TGF-β signaling is involved in normal epidermal development, maintenance of homeostasis [59], and regulation of the hair follicle cycle [60,61]. In summary, the more highly expressed gene sets in the fine wool group in these pathways play key roles in regulating hair structure.

To further explore the function of DEGs, PPI analysis was performed, and hub nodes and genes with a high degree of connection involved in key pathways were filtered. *EGFR* plays an important role in the control of epithelial hair layer development and hair shape [62]. Mice display a pronounced delay in hair follicle development when they completely lack *EGFR* [63]. *VEGFA* is paracrine factor that contributes to hair follicle growth and cell cycle regulation and stimulates the proliferation of dermal papilla cells [64,65]. The regulatory network also showed that DEGs including *LEF1*, *FZD3*, *SMAD3*, and *BMP4* were important regulators of the Wnt signaling, basal cell carcinoma, and Hippo signaling pathways. *NOTCH2*, *MAML3*, and *DTX4* were substantially enriched in Notch signaling. *ITGB6* was also differentially regulated in ECM–receptor interaction between the two groups. These findings filter the crucial functional DEGs further.

## 5. Conclusions

This study identified differences in the characterization of coarse and fine wool and their hair follicle traits, as well as the transcriptome profiles of hair follicles of coarse and fine wool from Wan strain Angora rabbits. The morphological and histological analysis showed that the diameters of coarse wool and hair follicles were substantially larger than those of fine wool. The mRNA profile analysis obtained potential functional genes and pathways regulating hair structure. Further studies are required to investigate the roles of candidate functional genes that regulate hair structure to improve super-fine wool rabbit breeding programs.

## Figures and Tables

**Table 1:** Expression levels of differentially expressed KRT and KAP genes between coarse and fine wool of Angora rabbits.

Gene_ID	FPKM Value		log_2_FoldChange	padj	Gene_Name
	CW	FW	C/F		
ENSOCUG00000008097	12,772.98946	50,873.64262	−1.993831042	8.23 × 10^−36^	KRT72
ENSOCUG00000029195	4355.248887	11,770.33261	−1.43434679	4.32 × 10^−10^	KRT73
ENSOCUG00000000501	1593.270426	6539.650031	−2.037557203	1.41 × 10^−19^	KRT82
ENSOCUG00000013084	13,194.53043	31,919.36712	−1.274436947	2.83 × 10^−16^	KRT35
ENSOCUG00000002607	32,696.88974	87,967.21994	−1.427803688	3.34 × 10^−12^	KRT85
ENSOCUG00000013079	6008.189844	13,499.59989	−1.167961219	8.47 × 10^−5^	KRT32
ENSOCUG00000014195	32,907.09207	74,808.91002	−1.184801978	1.53 × 10^−4^	KRT34
ENSOCUG00000015593	6515.985431	15,504.91326	−1.250664324	2.25 × 10^−4^	KRT39
ENSOCUG00000031178	2.622223852	14.45444295	−2.48452346	3.92 × 10^−2^	KRT77
ENSOCUG00000015615	42,284.08017	205297.44	−2.279528889	2.97 × 10^−13^	KRTAP3-1
ENSOCUG00000025222	1890.382146	4169.785928	−1.141019559	2.40 × 10^−5^	KRTAP17-1
ENSOCUG00000029569	41,948.67877	11,894.10929	1.818463967	2.19 × 10^−27^	KRT17
ENSOCUG00000011731	194,355.7353	81,574.58156	1.25250161	9.47 × 10^−21^	KRT10
ENSOCUG00000008992	427548.044	155,569.5872	1.458521344	2.19 × 10^−27^	KRT25
ENSOCUG00000027870	11,282.85925	1317.991488	3.098259513	3.83 × 10^−20^	KRTAP6-1
ENSOCUG00000029234	486,944.5473	152,777.5888	1.67232842	1.66 × 10^−36^	KRT71
ENSOCUG00000017421	27,979.26417	11,240.11345	1.315757804	1.88 × 10^−6^	KRT5

**Table 2:** Gene sets enriched in the hair follicle of fine wool group.

Gene Set Name	NES	NOM *p*-Value
ECM–receptor interaction	−1.6106249	0
Wnt signaling pathway	−1.5830796	0
Basal cell carcinoma	−1.5305877	0
TGF-beta signaling pathway	−1.5286138	0
Notch signaling pathway	−1.5271446	0
Hippo signaling pathway	−1.51096	0

## Data Availability

The data are presented in this study and the Supplementary Materials. The sequencing data have been deposited in the Short Read Archive (SRA) under the accession number SRP415214 and BioProject accession number PRJNA916820.

## Supplementary Materials

The following supporting information can be downloaded at: https://www.mdpi.com/article/10.3390/biology12030445/s1, Table S1: Primers for qPCR. F1, forward primer. R2, reverse primer; Table S2: Summary of RNA-Seq data for each sample; Table S3: The significant GO terms of differentially expressed genes; Table S4: The core genes identified in ECM–receptor interaction by GSEA; Table S5: The core genes identified in Wnt signaling pathway by GSEA; Table S6: The core genes identified in basal cell carcinoma by GSEA; Table S7: The core genes identified in TGF-β signaling pathway by GSEA; Table S8: The core genes identified in Notch signaling pathway by GSEA; Table S9: The core genes identified in Hippo signaling pathway by GSEA; Figure S1: The DEGs in hair follicles of coarse and fine wool from Wan strain Angora rabbits. Red bar represents the number of up-regulated DEGs, green bar represents the number of down-regulated DEGs.
