# Supplementary material for: Hair Follicle Transcriptome Analysis Reveals Differentially Expressed Genes That Regulate Wool Fiber Diameter in Angora Rabbits
Source: Biology (Basel). 2023 Mar 14;12(3):445. doi: 10.3390/biology12030445 (PMC10045444; doi:10.3390/biology12030445)
Supplement: Supplementary file 1 [file biology-12-00445-s001.zip › Supplementary Figure.pdf]

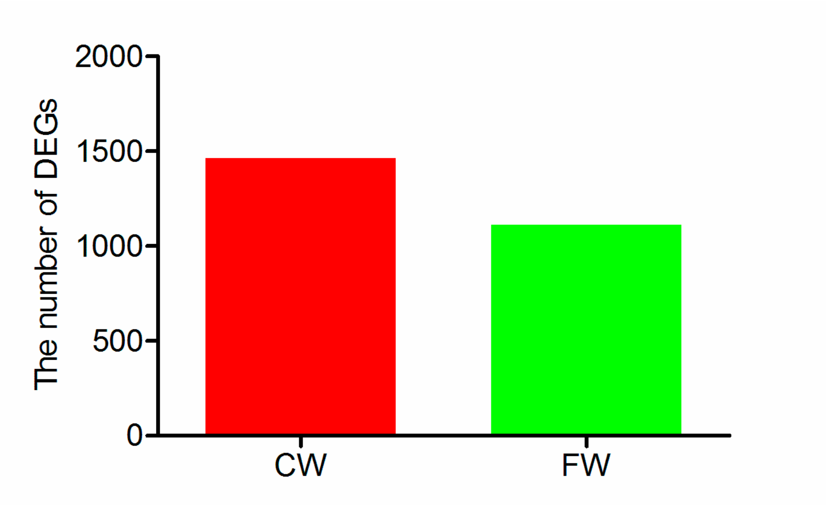


**Figure S1.** The DEGs in hair follicles of the coarse and fine wool from Wan strain Angora rabbits. Red bar represents the number of up-regulated DEGs, Green bar represents the number of down-regulated DEGs.
